# Pediatric Cardiac Arrest Outcomes in the United States: A Nationwide Database Cohort Study

**DOI:** 10.7759/cureus.26505

**Published:** 2022-07-01

**Authors:** Tanveer Mir, Obeid M Shafi, Mohammad Uddin, Meghana Nadiger, Fnu Sibghat Tul Llah, Waqas T Qureshi

**Affiliations:** 1 Internal Medicine, Wayne State University Detroit Medical Center, Detroit, USA; 2 Pediatrics, Arkansas Children's Hospital, Little Rock, USA; 3 Clinical Informatics, University of Arkansas for Medical Sciences, Little Rock, USA; 4 Pediatrics, Herbert Wertheim College of Medicine, Miami, USA; 5 Pediatric Critical Care Medicine, Nicklaus Children's Hospital, Miami, USA; 6 Cardiology, Jack Stephens Institute, Little Rock, USA; 7 Cardiology, University of Massachusetts, Worcester, USA

**Keywords:** outcomes, inpatient, emergency department, survival rate, nationwide emergency database sample, cardiopulmonary resuscitation, pediatric cardiac arrest

## Abstract

Background

Knowledge about the causes and outcomes of pediatric cardiac arrest in the emergency department is limited. The aim of our study was to evaluate the characteristics and outcomes of pediatric cardiac arrest in the emergency department (EDCA) and inpatient (IPCA) settings in the United States using a large database designed to provide nationwide estimates.

Methods

We performed a retrospective cohort study using the Nationwide Emergency Department Sample (NEDS), a database that includes both ED and inpatient encounters. The NEDS was analyzed for episodes of cardiac arrest between 2016-2018 in patients aged ≤18 years. Patients with cardiac arrest were identified using the International Classification of Diseases, 10th revision codes.

Results

A total of 15,348 pediatric cardiac arrest events with cardiopulmonary resuscitation were recorded, of which 13,239 had EDCA and 2,109 had IPCA. A lower survival rate of 19% was observed for EDCA compared to 40.4% for IPCA. While more than half of the EDCA events had no associated diagnoses, trauma (15.6%), respiratory failure (5%), asphyxiation (2.7%), acidosis (2.4%), and ventricular arrhythmia (1.4%) were associated with the remaining events. In comparison, the most frequently associated diagnoses for IPCA were respiratory failure (75.8%), acidosis (43.9%), acute kidney injury (27.2%), trauma (27.1%), and sepsis (22.5%).

Conclusions

Survival rates for EDCA were less than half of that for IPCA. The low survival rates along with the distinctive characteristics of EDCA events suggest the need for further research in this area to identify remediable factors and improve survival.

## Introduction

It is estimated that more than 20,000 children have a cardiac arrest (CA) annually in the United States (US) [1-4]. Pediatric CA requiring resuscitation with chest compression and/or defibrillation can be classified as a pulseless event or as a non-pulseless event such as bradycardia leading to poor perfusion [5]. Approximately 15,000 children receive cardiopulmonary resuscitation (CPR) for in-hospital cardiac arrest (IHCA) annually [1]. In 2015, the incidence of IHCA in children was approximately 1.89 events per 1000 inpatient days and 12.66 events per 1000 admissions [4]. The overall incidence rate for out-of-hospital cardiac arrest (OHCA) is 8.3 per 100,000 person-years [6]. In comparison to OHCA, pediatric IHCA has higher rates of survival [6-8]. 

There are limited national-level studies regarding the outcomes of emergency department cardiac arrest (EDCA) in comparison to IHCA for the pediatric population within the US. IHCA events in pediatric patients differ substantially from adults [7,9,10]. The majority of pediatric IHCA (85-90%) occur in monitored settings, often in the backdrop of progressive shock or respiratory failure [4,7,11-13]. While outcomes for pediatric IHCA improved during the first decade of the 21^st^ century, the survival rates have plateaued since 2010 [4,5,14]. The overall survival rates for pediatric IHCA were 41.1% compared to 11.4% for OHCA [4,5,14]. Despite higher survival rates of IHCA, more national-level studies are needed to further assess the clinical characteristics and outcomes for CA at different locations within the hospital [14,15]. Worldwide, studies of pediatric CA in the emergency department (ED) have reported survival rates between 12.8 to 33.8% [16-19]. 

Previously, studies have evaluated the survival of pediatric patients as IHCA and OHCA; however, no studies have analogized the clinical outcomes of inpatient cardiac arrest (IPCA) and EDCA. Assessing the prevalence, predictors, and causes of pediatric CA based on location within a hospital would allow for a better understanding of this modifiable disease process to help improve outcomes and healthcare policy. Thus, we evaluated the characteristics and survival rates for pediatric cardiac arrest in the ED and inpatient settings from the Nationwide Emergency Department Sample (NEDS) database. 

## Materials and methods

Study population and inclusion criteria

This is an observational cohort study of patients with cardiac arrest in the Nationwide Emergency Department Sample (NEDS) database between 2016-2018. The NEDS is the largest all-payer ED database that is publicly available in the United States and has in-hospital encounters, which include ED encounters and inpatient encounters. Since the database constitutes approximately 20% of the population, a weighted analysis is recommended to generalize it for the entire population of the US, with unweighted estimates considered invalid [20]. Thus, all NEDS estimates were calculated using national weighted values and accounted for sample weighting. Using a stratified, random sampling design, a sample of hospital-owned emergency departments from the US participating in both the State Inpatient Databases (SID) and the State Emergency Department Databases (SEDD) was selected. One hundred percent of the emergency department visits and inpatient encounters from the selected emergency departments were retained for the study dataset. The SEDD captures information on ED visits that do not result in an admission, while the SID contains information on patients initially seen in the emergency and then admitted to the hospital [20]. 

Hospitals were included in the NEDS sample based on geographic region, teaching status (non-teaching or teaching), location (urban or rural), ownership (public, private for-profit, private not-for-profit), and trauma center designation. A total of 990 emergency departments were included in the NEDS database. From each selected emergency department, all visits were included, which amounted to more than 33 million unweighted visits each year. Weighted, the database estimates more than 140 million ED visits and more than 20 million inpatient admissions per year. The institutional review board declared this study exempt from review. However, it was performed according to the ethical criteria set up by Healthcare Cost and Utilization Project (HCUP) [20]. 

Study definitions

Cardiac arrest was defined by International Classification of Diseases-10 (ICD-10) codes of I46.2, I46.8, I46.9, I97.121, and I97.11. We excluded patients older than 18 years. We divided patients into EDCA and IPCA based on the site where CPR was performed. Patients who had a CA in the emergency department (EDCA) were identified with "current procedural terminology codes (CPT)" for cardiopulmonary resuscitation as ED procedures are added to the database as CPT codes. Patients who had a CA in the inpatient (IPCA) setting were identified using "procedure codes (PR)" for CPR as the inpatient sample does not have CPT codes (supplemental file, section II). This method has been previously validated and published in the literature [21]. Patient demographics, payer information, and comorbidities were obtained from the database. The information about diagnoses associated with CA was defined by the ICD-10 codes using the links provided in the appendix.

Patient and hospital characteristics

Baseline patient demographic characteristics (age, sex, insurance payer, hospital location) were extracted using database variables. Diagnostic codes were used to identify comorbidities of hypertension, congenital heart disease, malignancy, diabetes mellitus, hyperlipidemia, obesity, asthma, epilepsy, sickle cell disease, and renal failure. Since HCUP databases cannot evaluate causation and can only check for an association, we extracted data for variables that have been related as causes and contributing factors by previously published studies, which included asphyxiation and strangulation, cardiac surgery, ventricular tachycardia/fibrillation, shock, acidosis, sepsis, trauma, respiratory failure, hyperkalemia, cerebral edema, pneumonia, cardiomyopathy, hypothermia, bronchiolitis, pneumothorax, acute kidney injury, and pulmonary embolism [7,19,22,23].

Outcomes

The primary outcome of the study was survival to discharge. We also studied the characteristics associated with CA at different locations.

Statistical methods

Categorical variables were expressed as weighted values and percentages. Continuous variables were expressed as mean ± standard deviation if the variable was not skewed; otherwise, it was expressed as a median with 25^th^ and 75^th^ percentiles (interquartile range). Descriptive statistics were performed for demographics and comorbidities, which were stratified by the type of CA (IPCA and EDCA). We used survey statistics to calculate Pearson's chi-square test for categorical variables and the t-test for the continuous variables. We also evaluated the associated diagnoses and provided weighted values along with their percentages for the subgroups of patients that had CA in the emergency department and inpatient setting. The proportions were compared using Pearson's chi-square test. All analyses were weighted analyses. Statistical analysis was performed using STATA version 16.1 (College Station, USA). Using STATA 16.1, we confirmed that there was no overlap in our cohort of patient encounters for those who had a cardiac arrest in the emergency department and those with cardiac arrest in the inpatient setting. All p-values were two-sided, with a significance threshold of p<0.05. 

## Results

**Figure 1:**
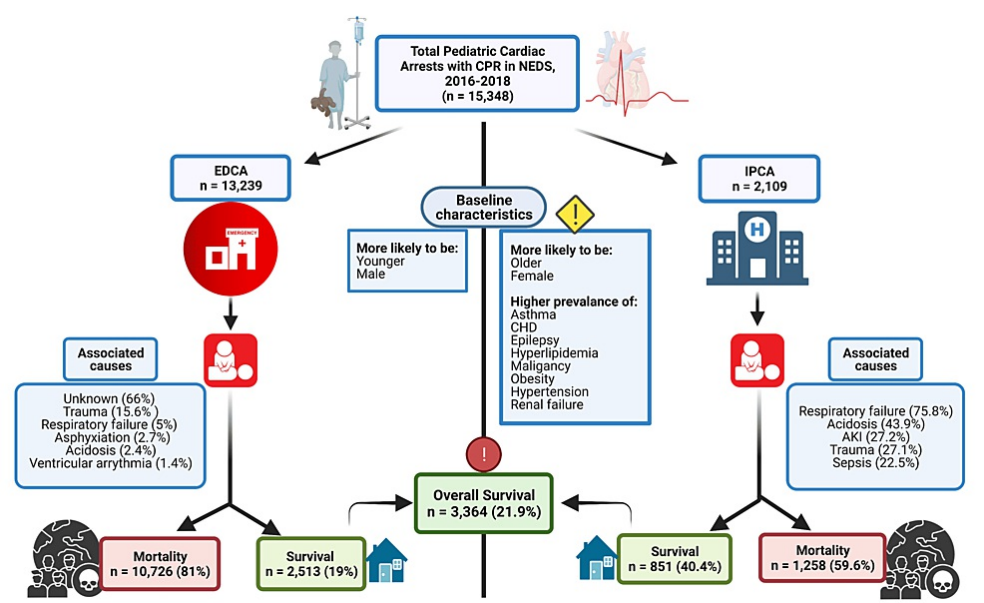
Graphical abstract of the analysis AKI - acute kidney injury; CHD - congenital heart disease; CPR - cardiopulmonary resuscitation; EDCA - emergency department cardiac arrest; IPCA - inpatient cardiac arrest; NEDS - Nationwide Emergency Department Sample Image created with BioRender.com.

A total of 34,763 pediatric CAs were recorded in the NEDS database for the years 2016-2018. Of the 15,348 patients (mean age 4.8±6 years, 39.9% females) who had CPR, 13,239 had EDCA, and 2,109 had IPCA (Figure 1).

The mean age of patients with EDCA was 4.5±5.9 years, compared to 6.2±6.2 years for IPCA (p<0.001). The proportion of males was 60.8% and 56.6% in the ED and inpatient groups (p=0.044), respectively. The baseline characteristics for both groups are compared in Table 1. The proportion of patients with comorbidities of asthma, congenital heart disease, epilepsy, hyperlipidemia, obesity, hypertension, malignancy, and renal failure were all more common in the group with IPCA. Medicaid was the predominant payer among all CA patients.

**Table 1:** Baseline characteristics of patients with EDCA and IPCA EDCA - emergency department cardiac arrest; IPCA - inpatient cardiac arrest

Variables, n (%)	EDCA (n=13,239)	IPCA (n=2,109)	p-value
Primary payer			
Medicare	392 (2.9%)	79 (3.8%)	<0.001
Medicaid	7,608 (57.5%)	1,253 (59.4%)	<0.001
Private insurance	3,270 (24.7%)	699 (33.1%)	<0.001
Self-pay	1,969 (14.9%)	78 (3.7%)	<0.001
Hospital characteristics			
Teaching hospital	7,933 (59.9%)	2,053 (97.4%)	<0.001
Urban location	11,969 (90.4%)	1,907 (90.5%)	0.986
Pre-existing conditions			
Asthma	324 (2.5%)	215 (10.2%)	<0.001
Congenital heart disease	289 (2.18%)	293 (13.9%)	<0.001
Diabetes mellitus	57 (0.4%)	30 (1.4%)	0.077
Epilepsy	192 (1.5%)	138 (6.5%)	<0.001
Hyperlipidemia	10 (0.06%)	18 (0.8%)	0.049
Hypertension	58 (0.4%)	231 (11%)	<0.001
Malignancy	23 (0.17%)	56 (2.7%)	0.002
Obesity	11 (0.06%)	19 (0.9%)	0.043
Renal failure	32 (0.24%)	34 (1.6%)	0.032
Sickle cell disease	49 (0.37%)	20 (0.9%)	0.207

Survival 

The overall survival to discharge rate for patients with CA undergoing CPR was 21.9% (n=3,364) during the study period. Of the patients who had EDCA, 2,513 (19%) survived post-CPR to discharge. Among IPCA patients, 851 (40.4%) survived post-CPR to discharge.

Characteristics associated with cardiac arrest

Eight thousand eight hundred and four patients with EDCA had no evidently associated diagnosis. Among those EDCA patients with an associated diagnosis, trauma was the predominant diagnosis, accounting for 15.6% of patients. Others were respiratory failure (5.01%), asphyxiation and strangulation (2.7%), acidosis (2.4%), ventricular arrhythmia (1.4%), hyperkalemia (1.4%), hypothermia (1.2%) and sepsis (1.1%). In contrast, the most frequently associated factors among IPCA were respiratory failure (75.8%), acidosis (43.9%), acute kidney injury (27.2%), trauma (27.1%), sepsis (22.5%), pneumonia (20.7%), shock (19%), cerebral edema (13.6%), ventricular tachycardia/fibrillation (12.9%), and hyperkalemia (11.1%). Conditions associated with EDCA and IPCA are presented in Table 2.

**Table 2:** Conditions associated with EDCA and IPCA Since cardiac arrest can be multifactorial, the percentages do not add up. EDCA - emergency department cardiac arrest; IPCA - inpatient cardiac arrest

Variables, n (%)	EDCA (n=13,239)	IPCA (n=2,109)	p-value
Acidosis	320 (2.4%)	926 (43.9%)	<0.001
Acute kidney injury	28 (0.21%)	573 (27.2%)	<0.001
Asphyxiation and strangulation	357 (2.7%)	49 (2.3%)	0.606
Bronchiolitis	17 (0.13%)	201 (9.5%)	<0.001
Cardiac surgery	11 (0.07%)	52 (2.5%)	<0.001
Cardiomyopathy	22 (0.17%)	64 (3%)	<0.001
Cerebral edema	31 (0.23%)	287 (13.6%)	<0.001
Hyperkalemia	181 (1.4%)	233 (11.1%)	<0.001
Hypothermia	157 (1.2%)	43 (2%)	0.129
Pneumonia	106 (0.8%)	436 (20.7%)	<0.001
Pneumothorax	104 (0.8%)	138 (6.5%)	<0.001
Pulmonary embolism	10 (0.06%)	131 (6.2%)	<0.001
Respiratory failure	663 (5.01%)	1,598 (75.8%)	<0.001
Sepsis	140 (1.1%)	474 (22.5%)	<0.001
Shock	21 (0.2%)	393 (19%)	<0.001
Trauma	2,059 (15.6%)	571 (27.1%)	<0.001
Ventricular tachycardia/fibrillation	186 (1.4%)	272 (12.9%)	<0.001

## Discussion

This study, based on our literature review, is the first study to have evaluated the different characteristics and survival rates of pediatric patients who had cardiac arrest based on their location in the ED or inpatient setting. While previous studies have looked at attributes of OHCA and IHCA, our study explored attributes of pediatric EDCA and IPCA. The most salient features of our study were: (a) survival was lower for patients with EDCA, and (b) while more than half of the patients with EDCA did not have another primary diagnosis, trauma was the most frequently observed associated diagnosis in the remaining patients. In contrast, patients with IPCA were commonly observed to have disorders of respiratory failure, acidosis, acute kidney injury, sepsis, and shock, in addition to trauma. 

Survival

We observed an overall survival to discharge rate of 21.9%. There was a significant difference in rates of survival based on location, with pediatric IPCA patients (40.4%) having more than twice the survival rates compared to those with EDCA (19%). Since no previous studies have looked at survival rates for pediatric CA based on their location like our study, it is difficult to make precise comparisons. 

The rates of survival to discharge for pediatric patients with CA in the inpatient setting (40.4%) agree closely with findings reported in the 2020 update from the American Heart Association (AHA) for pediatric IHCA (41.1%) [4]. Other studies have also looked at the trends in the survival rates for pediatric CA. Girotra et al., in their 2013 study, using the Get With The Guidelines-Resuscitation (GWTG-R) registry, described survival rates of 14% and 39% for pediatric IHCA in 2000 and 2009, respectively [14]. A more recent study utilizing the same GWTG-R registry reported rates for survival to discharge of 38% and 66% in 2018 for pediatric IHCA patients with pulseless and non-pulseless CA, respectively [5]. Virani et al., in their 2020 report for the AHA, delineated 41.1 % survival rates for children with IHCA [4]. Although the survival rates have increased by 0.67% per year on average between 2000 to 2018, there has been a plateau in the increase of survival rates since 2010, hence the need for continued focus, research, and improvement in this area [5]. Skellett et al. reported an overall survival to discharge rate of 54.2% after pediatric in-hospital cardiac arrest in the United Kingdom from their seven-year review of the National Cardiac Arrest Audit (NCAA) database [24]. 

There is a paucity of literature on rates of survival for pediatric patients with EDCA. Michelson et al., in a retrospective study using the NEDS, established a survival rate of 21.7% for pediatric cardiac arrest in the ED for the years 2009-2014. For non-traumatic CAs, they reported survival rates of 33.8% in the pediatric ED versus 18.9% in the general ED. In their study, the survival rates for traumatic OHCA were 31.7% and 26.1% in pediatric and general EDs, respectively [19]. Our findings of a survival rate of 19% in patients with EDCA are like the overall rate in the study by Michelson et al. (21.7%), where notably, the same NEDS database was utilized [19]. Donoghue et al., utilizing the GWTG-R registry, reported a survival to discharge rate of 20% for pediatric patients with CPR initiated in the ED [17]. Another study reported survival rates of 12.8% in children with CA between 2008-2012 using the Korean ED-based clinical registry [16]. A single-center, retrospective study from Turkey observed a survival rate of 18% for cardiac arrest in a tertiary-center pediatric ED over a period of three years [18]. 

The higher survival rates for IPCA compared to EDCA could stem from a myriad of reasons, including early recognition in a monitored setting, greater availability of experienced staff, better preparedness, and known etiology of CA. The disparate nature of EDCA, where some OHCA events may have been included in the analysis, could also contribute to lower survival rates for this location in our study. 

Characteristics associated with cardiac arrest 

The causes for cardiac arrest in pediatrics are numerous and include respiratory, traumatic, infectious, and cardiac conditions. Pertinently, pediatric CA is less likely to be a primary cardiac event in comparison to adults [25]. In our study, trauma was recorded as the most associated factor for EDCA events, reported in 15.6% of patients. Michelson et al. reported trauma to be associated in around 25% (24.9% to 28.6%) of patients with EDCA [19]. Other characteristics associated with this group included respiratory failure, asphyxiation and strangulation, and acidosis. In contrast, pediatric IHCA is often caused by progressive respiratory insufficiency and/or shock [7,11,12]. The most frequently associated characteristics with IPCA in our study were also respiratory failure, acidosis, acute kidney injury, trauma, sepsis, and shock. 

Acidosis has been frequently observed in patients with CA and is likely due to a combination of metabolic abnormalities, including increased lactate levels, unmeasured anions, and acute kidney injury, as well as respiratory insufficiency [26]. Acidosis is an important contributing factor and complication of CA, as acidemia impairs myocardial function and diminishes myocardial response to catecholamines [23,27]. In addition, acidosis and sepsis are directly related to each other and worsen the mortality outcomes [28]. The high rates of sepsis and acidosis observed in our study suggest that clinicians should remain vigilant for early identification and proper management of these likely reversible contributing factors of CA. 

Interestingly, the EDCA patients had fewer comorbidities than IPCA but higher mortality rates. Since almost one-sixth of EDCA patients had trauma as the associated diagnosis, they could potentially have been previously healthy. This could also suggest that the patients with more comorbidities get triaged quicker to perform a lifesaving intervention, or it could reflect the under-evaluation of patients with EDCA. This emphasizes the need to further evaluate the other predictors of EDCA in pediatric patients. 

Important strengths of this study are the large number of cases on which estimates are based and its national representativeness. However, the study also has certain limitations. Since the study is a retrospective, observational study, inference regarding causation should be made with caution. The NEDS database can show association but can not imply causation or provide temporal event data. Additionally, the NEDS registry is unable to definitively differentiate in-ED cardiac arrests from OHCA and only includes information for inpatients that are admitted through the ED. Also, we relied on reported ICD-10 codes to identify diagnoses to perform our analysis, which may not accurately reflect the true cause of cardiac arrest. The NEDS is an administrative database that could be subject to inaccurate coding and underreporting of comorbid diagnoses. There is an absence of valuable information related to patients' physical examination, electrocardiography, echocardiography, medications, laboratory results, radiologic findings, and long-term complications. Also, since the NEDS contains encounter-level records, a single patient may be represented multiple times in the database for different ED visits. However, the NEDS and the codes used in this study have been used in multiple clinical studies and can be considered a reliable database, given the large cohorts analyzed.

## Conclusions

In a nationally representative cohort from the NEDS database, over one-fifth of pediatric cardiac arrest patients undergoing CPR survived to hospital discharge. The survival for EDCA events was less than half of the rates for IPCA events. While trauma was a notably associated diagnosis in both settings, respiratory failure, acidosis, acute kidney injury, and sepsis were also commonly associated factors for the inpatient location. The low survival rates for EDCA suggest the need for further research to identify remediable factors and potentially improve survival. 

## Appendices

Supplemental information for identification of ICD codes, and procedure codes

Section I

Codes used for generating cardiac arrest files. All codes were ICD-10 codes.

I46.2, I46.8, I46.9, I97.121, and I97.11.

Section II

1) CPT codes used for procedures in the emergency department.

Procedure codes for cardiopulmonary resuscitation in the emergency department. (cpt1-cpt15)

92950

2) PR codes used for procedures in the in-patient setting.

Procedure codes for cardiopulmonary resuscitation for in-hospital cardiac arrest. (i10_pr_ip1-i10_pr_ip15)

5A12012, 02QA0ZZ

Codes used for the comorbidities were obtained from the following websites:

1) https://www.icd10data.com/

2) https://www.icd10data.com/ICD10PCS/Codes

3) https://icdcodelookup.com/icd-10/codes

4) https://www.cms.gov/Medicare/Coding/ICD10/
